# Robotic gastric Roux-en-Y bypass with or without concomitant cholecystectomy: pilot open label randomized trial

**DOI:** 10.1093/bjsopen/zrag092

**Published:** 2026-08-18

**Authors:** Marie Burgard, Marco Bonino, Marion Dietrich, Pouya Iranmanesh, Michael Racine, Guillaume Giudicelli, Stefan P Mönig, Christian Toso, Minoa K Jung

**Affiliations:** Department of Visceral Surgery, HUG–Hôpitaux Universitaires de Genève, Geneva, Switzerland; Department of Visceral Surgery, HUG–Hôpitaux Universitaires de Genève, Geneva, Switzerland; Department of Visceral Surgery, HUG–Hôpitaux Universitaires de Genève, Geneva, Switzerland; Department of Visceral Surgery, HUG–Hôpitaux Universitaires de Genève, Geneva, Switzerland; Department of Visceral Surgery, HUG–Hôpitaux Universitaires de Genève, Geneva, Switzerland; Department of Visceral Surgery, HUG–Hôpitaux Universitaires de Genève, Geneva, Switzerland; Department of Visceral Surgery, HUG–Hôpitaux Universitaires de Genève, Geneva, Switzerland; Department of Visceral Surgery, HUG–Hôpitaux Universitaires de Genève, Geneva, Switzerland; Department of Visceral Surgery, HUG–Hôpitaux Universitaires de Genève, Geneva, Switzerland

## Abstract

**Background:**

Debate continues about the need for prophylactic concomitant cholecystectomy (CC) at the time of Roux-en-Y gastric bypass (RYGB). The concerns over higher complication rates should be balanced against the risk of future biliary events. This pilot trial compared RYGB with CC to RYGB alone in patients without preoperative gallstones to inform a future large randomized trial.

**Methods:**

Eligible patients without gallstones scheduled to undergo RYGB were randomized before surgery using a 1 : 1 allocation ratio. Surgeons and patients were not blinded. The primary outcome was a composite of intraoperative adverse events and 30-day postoperative complications. In addition, feasibility (time to enrolment, completion of follow-up) was added as a co-primary endpoint after the study started, driven by prolonged enrolment times and independent of trial results. Secondary outcomes included late complications, biliary events, length of operation, length of hospital stay, and quality of life.

**Results:**

In all, 96 patients were randomized between July 2018 and January 2023, and 95 were included in the full analysis due to one withdrawal. There were 49 and 46 patients in the RYGB with and without CC arms, respectively. The feasibility endpoint was met with regard to the completion of follow-up (95/95, 100% at 1 month; 88/95 93% at 18 months), but not the time to enrolment (21 patients randomized per year). The composite outcome of intraoperative and postoperative complications occurred in 44.9% and 26.1% of patients with and without CC, respectively (difference 18.8%; 95% confidence interval (c.i.) −0.6 to 36.8; *P* = 0.098). Patients with CC had a higher early comprehensive complication index score (mean 6.09 versus 2.06; difference 4.03; 95% c.i. 0.23 to 7.83; *P* = 0.047). Late (> 30 days) complications, length of hospital stay, and quality of life were similar between the two groups. During the 18-month follow-up, biliary events occurred in 3 patients without CC (6.5%), of whom 2 required biliary intervention (4.4%) compared with 1 patient with CC (2%), with no biliary interventions required.

**Conclusion:**

The debate over performing CC during RYGB remains unresolved. The incidence of subsequent biliary interventions was low when CC was not performed. As a pilot study, this investigation sets the stage for a large randomized trial. Registration number: NCT04324515 (https://www.clinicaltrials.gov).

## Teaser

This pilot trial evaluated the impact of prophylactic concomitant cholecystectomy (CC) during Roux-en-Y gastric bypass in patients without preoperative gallstones, finding that CC increased postoperative complications. Although CC did not reduce the incidence of biliary events, the study's feasibility results support the need for a larger randomized trial to further assess these findings.

## Introduction

Weight loss is a well known risk factor for the development of gallstone disease, particularly when the weight loss is significant. This is especially true in the context of metabolic and bariatric surgery (MBS)^1^. Indeed, approximately 20% of patients who undergo MBS develop new gallstones in the first 12 months after the procedure^2,3^, and there is a fivefold increased risk of developing symptomatic gallstone disease after MBS compared with the general population^4^. In addition, the altered postoperative anatomy, especially after Roux-en-Y gastric bypass (RYGB), complicates the management of gallstone migration, because endoscopic management is not feasible without accessing the excluded stomach. These challenges raise questions about gallbladder management in patients undergoing MBS, particularly regarding the role of prophylactic cholecystectomy.

The literature offers mixed results on concomitant cholecystectomy (CC). Some studies indicate a higher complication rate for MBS when combined with than without cholecystectomy^3,5,6^, whereas other studies report similar safety profiles between the two approaches^7,8^.

Although gallstone development is more common among patients who have undergone MBS, subsequent cholecystectomy for symptomatic gallstone disease is performed in only 2–9% of patients^3,4,9,10^, and the incidence of symptomatic gallstones is higher in patients with gallstones detected at preoperative ultrasound^3,10^.

There are currently four approaches to the management of the gallbladder in patients undergoing MBS: routine prophylactic CC in all patients undergoing MBS; selective prophylactic CC for all patients with gallstones at preoperative ultrasound; selective CC only for symptomatic patients; and prophylactic medical treatment with ursodeoxycholic acid. Many surgeons recommend a selective approach^11^, but there are no established guidelines, and randomized clinical trials are lacking.

Owing to the uncertainty regarding whether CC provides a benefit or causes harm in patients undergoing MBS, a large, adequately powered randomized trial is warranted. Before initiating such a trial, the aim of the present study was to assess its feasibility in terms of time to enrolment and completion of follow-up. To obtain a homogeneous study population, this study focused on patients without gallstones on preoperative ultrasound. In addition, the study aimed to examine preliminary endpoints, especially a composite of intraoperative adverse events and early postoperative complications, to provide a foundation for future study design.

This pilot randomized trial compared RYGB with and without CC in patients without evidence of gallstones at preoperative ultrasound.

## Methods

### Study design and participants

This was a pilot open-label randomized parallel two-arm trial, conducted at a bariatric surgery unit of a single tertiary care academic university hospital, comparing RYGB with CC (study group) to RYGB without CC (control group) in patients without gallstones on preoperative ultrasound. Because this was a pilot study, superiority, non-inferiority, and equivalence were not tested. Patients eligible for inclusion were those aged ≥ 18 years who met the criteria of the Swiss Society for the Study of Morbid Obesity to undergo gastric bypass^12^, namely a body mass index (BMI) of ≥ 35 kg/m^2^, having followed a 2-year programme for weight loss without success (or a 1-year programme in the case of BMI > 50 kg/m^2^), and who consented to multidisciplinary follow-up for 5 years.

Exclusion criteria included the following contraindications for gastric bypass according to the Swiss Society for the Study of Morbid Obesity: pregnancy, kidney failure (glomerular filtration rate <30 ml/min) without dialysis, cirrhosis Child B/C, ulcerative colitis, pulmonary embolism or deep venous thrombosis during the past 6 months, psychiatric contraindications, or drug abuse (alcohol, cannabis, opioids) during the past 6 months.

Furthermore, patients with previous cholecystectomy, gallstones or polyps on preoperative ultrasound, and those with a gallbladder that could not be assessed on ultrasound were excluded. Patients with important language barriers or participating in other studies were also excluded, as were those aged < 18 years. Patients with planned concomitant interventions (such as abdominal wall hernia repair requiring mesh placement), those undergoing revision surgery or sleeve gastrectomy, and those with steatosis diagnosed on ultrasound or elevated liver enzymes in preoperative blood samples were also excluded.

Patients were recruited from the principal investigator’s M.K.J. or co-investigators’ clinical practices. Potentially eligible patients were approached with the study information and were provided with written information. At the next visit, written informed consent was obtained.

The study was conducted in compliance with the trial protocol, the current version of the Declaration of Helsinki, the International Council for Harmonisation’s Good Clinical Practice or the international standard ISO EN 14155, and all national legal and regulatory requirements (Loi sur les produits thérapeutiques 812.21 and Ordonnance sur les essais cliniques des produits thérapeutiques 812.214.2). The study was approved by the local ethics committee Cantonal Ethics committee for Research (CCER) of the Geneva University Hospitals (Project ID 2016-01243). The trial report was based on the CONSORT guidelines^13^ and the CONSORT guidelines for feasibility trials^14^. The CONSORT checklist is provided in the *Supplementary material*.

The study’s original primary outcome was a composite of intraoperative adverse events and early postoperative complications. However, due to slower-than-expected enrolment, the protocol was revised, and a feasibility outcome was included as co-primary endpoint to guide the design of future research. This modification was made independently of the trial results.

The trial protocol is available in the *Supplementary material*.

### Randomization

Patients who met the inclusion criteria were randomized by the clinical research centre of the University Hospital of Geneva using computer-generated random numbers and permuted blocks of varying lengths. To ensure concealment of allocation, double-sealed opaque envelopes that were numbered sequentially were used.

The randomization envelopes were opened by the study staff at the appropriate time before the scheduling of gastric bypass after subject selection. The randomization assignment form contained information specifying whether the cholecystectomy would be performed during the RYGB procedure.

### Procedures

All patients underwent RYGB with the Da Vinci Robotic Surgical System (Da Vinci X^®^ Robotic Surgical System, Intuitive Surgical, Sunnyvale, CA, USA). One 12-mm robotic trocar, three 8-mm robotic trocars, and one 5-mm assistant trocar were placed. For the RYGB, a small gastric pouch (∼40–50 ml) was created using blue cartridge staplers. A standard robotic RYGB with a 75-cm biliary limb and a 150-cm antecolic alimentary limb was then performed with a hand-sewn gastrojejunal and jejunojejunal anastomosis using a single-layer running suture of Vicryl 2/0. Mesenteric and Petersen’s defects were closed using non-absorbable Ethibond 2/0 running sutures. For the CC group, the intervention ended with a robotic cholecystectomy, and no additional trocar was placed.

Preoperative demographic and clinical data were collected at baseline. Preoperative routine work-up included a physical examination, vital signs, laboratory analyses (haematology, chemistry, and HbA1c), sleep apnoea evaluation with polygraphy, abdominal ultrasound, upper gastrointestinal endoscopy, pulmonary function, preoperative anaesthesia consultation, and psychological evaluation. Intraoperative data were collected, including complications (for example, organ injury, bleeding, conversion), operative time, and estimated blood loss.

From surgery to discharge, the following patient information was collected: complications according to the Clavien–Dindo classification system^15^ and Comprehensive Complication Index (CCI); reoperation; time to reinitiation of nutrition; time to first bowel movement; time to opioid discontinuation; and length of hospital stay.

Follow-up assessments were performed at 1, 3, 6, 12, and 18 months after surgery to collect the following information: postoperative complications according to the Clavien–Dindo classification and CCI; reoperation; readmission; clinical and laboratory findings; and quality of life, assessed using the EQ-5D™ questionnaire^16^ at 1 and 12 months. In addition, at the 18-month follow-up, abdominal ultrasound was performed on all patients without CC.

Surgeons and patients were not blinded. However, outcome assessment was conducted with blinding; the surgeons involved in the follow-up evaluations were unaware of whether a cholecystectomy had been performed.

### Outcomes

The first co-primary outcome of this study was feasibility. This was assessed in terms of time to enrolment (number of participants randomized per month) and the follow-up rate at 1 and 18 months. The trial would be considered successful in terms of time to enrolment after randomization of 45 patients per year, enabling a reasonable time frame for a larger randomized trial. A follow-up of at least 95% of the patients at 1 month and 90% at 18 months would be considered successful.

The second co-primary outcome was a composite endpoint of intraoperative adverse events (common bile duct, liver, or digestive tract injuries, bleeding, conversion) and early (30-day) postoperative complications according to the Clavien–Dindo classification, including reoperation. The reported outcome is binary (yes/no). If any of the components were present (that is, any intraoperative adverse event or at least one postoperative complication (Clavien–Dindo I–V)), the outcome was considered met.

The secondary endpoints were intraoperative adverse events in detail, early and late (> 30 days) postoperative complications assessed by both the Clavien–Dindo classification and the CCI, operative time, length of hospital stay, readmission rate, quality of life at 1 and 12 months, weight loss, and pain (assessed using the echelle visuelle analogue scale) at 1, 3, 6, 12, and 18 months, biliary events during follow-up (defined as symptomatic gallstones, gallstone migration, cholecystitis, choledocholithiasis, cholangitis, biliary pancreatitis), and the presence of asymptomatic gallstones at the 18-month follow-up ultrasound. Quality of life was assessed using the EQ-5D™, which covers five dimensions: mobility, self-care, usual activities, pain/discomfort, and anxiety/depression. Pain intensity was evaluated using a visual analogue scale ranging from 0 (no pain) to 10 (worst imaginable pain). Weight loss was expressed as the percentage total weight loss calculated as ((initial weight − postoperative weight)/initial weight) × 100. Intraoperative haemorrhage was defined as any bleeding that required intervention through bipolar cautery or suturing. Gallstone migration was defined as the passage of a stone through the common bile duct, with no residual stones remaining in the bile duct. Discharge criteria encompassed the absence of fever (temperature ≤ 37.8°C), neutrophils ≤ 12 g/l, and the absence of anaemia (haemoglobin > 110 g/dl), nausea, or vomiting.

The trial was audited by a data safety monitoring board.

### Sample size

With a sample size of 45 participants in each arm, the prevalence of the composite outcome (adverse events and complications) would be assessed with a precision (that is, half the length of the 95% confidence interval (c.i.)) of ± 0.09 for a prevalence of 0.15 and ± 0.13 for a prevalence of 0.25.

### Statistical analysis

Feasibility outcomes are reported descriptively as numbers and percentages.

For the primary composite and secondary outcomes, the absolute risk difference with the two-sided 95% c.i. was calculated with the BinomDiffCI function in the R package DescTools (R statistical software, version 00.99.44, R core team, Vienna, Austria), whereas the mean difference with the Studentized two-sided 95% c.i. was computed using the basic R function t.test, with the Welch’s *t* test option. No correction on the level of confidence intervals for multiplicity was applied. Continuous variables with a strongly skewed distribution are reported as median values rather than the mean. The 95% confidence intervals were obtained with a bootstrap procedure (percentile approach) or, if the variability of the observations was small and invalidated the bootstrap procedure, with the Hodges–Lehman estimator. The χ^2^ test was used to test the association between the two categorical variables, with the level of significance defined as *P* < 0.05. Data were analysed using R.4.2.0 software (Foundation for Statistical Computing, Vienna, Austria; https://www.r-project.org).

The main analysis followed the intention-to-treat principle with a full analysis set. Nonetheless, one patient was excluded from the full analysis set because of post-randomization withdrawal.

An analysis following the per-protocol principle was conducted as a sensitivity analysis for the composite primary outcome. One patient (in addition to the patient excluded from the full analysis set) was excluded from the per-protocol analysis because they received the treatment assigned to the opposite study arm.

## Results

**Fig. 1:**
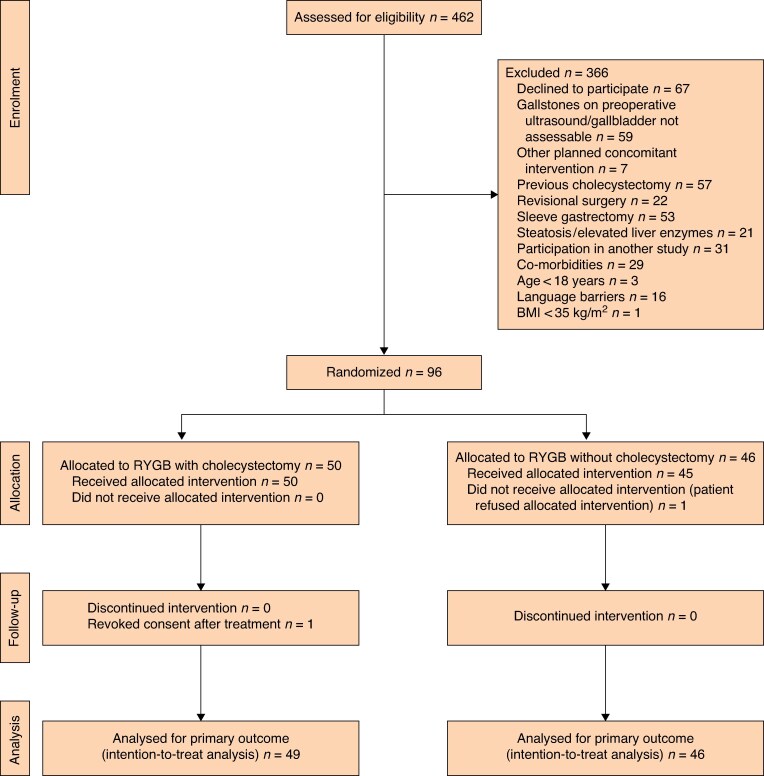
CONSORT flow diagram RYGB, Roux-en-Y gastric bypass; BMI, body mass index.

The recruitment period commenced on 1 July 2018 and ended on 20 January 2023. The follow-up period ran for 18 months and finished on 3 October 2024. *Figure 1* illustrates the study flow diagram. In total, 462 patients were screened for eligibility. After exclusion of patients meeting one or more exclusion criteria (366 patients), 96 patients were found to be eligible for the study and were randomized: 46 patients to RYGB without CC and 50 patients to RYGB with CC. One patient refused the allocated intervention (RYGB without CC) and was treated by RYGB with CC. One patient revoked consent after randomization, leaving 95 patients in the intention-to-treat analysis (46 in the control group, 49 in the study group).

### Baseline characteristics

The baseline characteristics were similar between the RYGB with and without CC groups (*Table 1*). The mean age of patients was 41 (range 19–67) years, and 83% were women. The mean BMI was 43.1 kg/m^2^. The most prevalent obesity-related complications were gastro-oesophageal reflux disease (49.5%), dyslipidaemia (49%), arterial hypertension (40%), sleep apnoea syndrome (37%), type 2 diabetes (21%), and hiatal hernia (20%).

**Table 1:** Demographic and clinical characteristics at baseline

	RYGB	
	Without cholecystectomy (*n* = 46)	With cholecystectomy (*n* = 49)
Age (years), mean(s.d.)	41.33(9.76)	40.80(10.62)
**Sex**		
Male	11 (23.9%)	5 (10.2%)
Female	35 (76.1%)	44 (89.8%)
Weight (kg), mean(s.d.)	121.73(22.77)	114.98(15.66)
BMI (kg/m^2^), mean(s.d.)	43.64(5.42)	42.58(5.15)
**ASA grade**		
I	0 (0%)	0 (0%)
II	23 (50%)	33 (67.3%)
III	23 (50%)	16 (32.7%)
IV	0 (0%)	0 (0%)
Tobacco use	0 (0%)	2 (4.1%)
**Co-morbidities**		
Hypertension	17 (37%)	21 (42.9%)
Coronary disease	3 (6.6%)	0 (0%)
Type 2 diabetes	10 (21.7%)	12 (24.5%)
Type 1 diabetes	2 (4.3%)	0 (0%)
Dyslipidaemia	16 (34.8%)	16 (32.7%)
Sleep apnoea syndrome	20 (43.5%)	15 (30.6%)
Thromboembolic disease	1 (2.2%)	0 (0%)
Chronic renal insufficiency	0 (0%)	2 (4.2%)
GERD	23 (50%)	24 (49%)
*Helicobacter pylori*	17 (37%)	19 (38.8%)
Hiatal hernia	9 (19.6%)	10 (20.4%)
Previous surgery	14 (30.4%)	23 (46.9%)

Values are *n* (%) unless otherwise stated. RYGB, Roux-en-Y gastric bypass; s.d. standard deviation; BMI, body mass index; ASA, American Society of Anesthesiologists; GERD, gastro-oesophageal reflux disease.

### Feasibility

In all, 96 patients were randomized in 55 months (21 patients randomized/year); thus, the predefined criteria for time to enrolment was not met. Group allocation was accepted by 99% of patients (94 of 95). Follow-up was completed at 1 month by 100% of patients (95), and at 18 months by 93% of patients (88 of 95).

### Intraoperative adverse events and early postoperative complications

*Table 2* presents detailed intraoperative adverse events and early postoperative complications in each group.

**Table 2:** Intraoperative adverse events and early (30 days) postoperative complications

	RYGB		Difference†	P*
	Without cholecystectomy *n* = 46	With cholecystectomy *n* = 49		
Composite primary endpoint‡	12 (26.1%)	22 (44.9%)	18.8 (−0.6, 36.8)	0.098
**Intraoperative events**	7 (15.2%)	7 (14.3%)	−0.9 (−16.1, 13.9)	0.831
Transfusion	0 (0%)	0 (0%)	0 (−7.8, 7.3)	
Biliary injury	0 (0%)	0 (0%)	0 (−7.8, 7.3)	
Liver injury	1 (2.2%)	1 (2%)	0 (−7.8, 7.2)	
Superficial intestinal injury	0 (0%)	4 (8.2%)	8.2 (0.1, 19.3)	
Haemorrhage	6 (13%)	2 (4.1%)	−9 (−22.3, 2.7)	
Conversion to open surgery	0 (0%)	0 (0%)	0 (−7.8, 7.3)	
Postoperative complications (30 days)	10 (21.7%)	17 (34.7%)	13.0 (−5.4, 30.5)	0.564
**Clavien–Dindo I**	9 (19.6%)	9 (18.4%)		0.814
Wound abscess (*n*)	4	3	−1.2 (−17.6, 14.9)	
Fever (*n*)	2	1	−2.6 (−15.2, 9.2)	
Bleeding (*n*)	0	1	−2.3 (−12.8, 6.9)	
Other (*n*)	3	4	2.0 (−5.8, 10.8)	
Wound bleeding (*n*)	2	1		
Electrolyte imbalance (*n*)	1	2		
Dysphagia (*n*)	0	1		
**Clavien–Dindo II**	1 (2.2%)	5 (10.2%)		0.120
Bleeding (*n*)	0	1	2 (−6, 10.6)	
Other (*n*)	1	4	7.1 (−7.1, 21.2)	
Hypertensive crisis (*n*)	0	1		
Vomiting (*n*)	0	1		
Skin infection (*n*)	0	1		
Allergic reaction (*n*)	0	1		
Urolithiasis (*n*)	1	0		
**Clavien–Dindo IIIa**	0 (0%)	1 (2%)		0.340
Anastomotic ulcer (*n*)	0	1		
**Clavien–Dindo IIIb**	0 (0%)	2 (4.1%)		0.175
Bleeding (*n*)	0	1	2 (−6, 10.6)	
Fistula (*n*)	0	1	2 (−6, 10.6)	
Clavien–Dindo IVa	0 (0%)	0 (0%)		
Clavien–Dindo V	0 (0%)	0 (0%)		
CCI score	6.09	2.06	4.03 (0.23, 7.83)	0.047
Reintervention	0 (0%)	2 (4%)	4 (−4.1, 13.5)	0.175

Values are *n* (%) unless otherwise stated. †Values in parentheses are 95% confidence intervals. ‡The primary endpoint was a composite of intraoperative adverse events and early postoperative complications. RYGB, Roux-en-Y gastric bypass; CCI, Comprehensive Complication Index. *χ² test.

In the intention-to-treat analysis, the composite primary endpoint occurred in 35.8% of all patients: 26.1% in the control group and 44.9% in the study group (difference 18.8%; 95% c.i. −0.6 to 36.8; *P* = 0.098). In the per-protocol analysis, the primary outcome occurred in 26.7% of patients in the control group and in 44.9% of in the study group (difference 18.2%; 95% c.i. −1.3 to 36.6; *P* = 0.083).

Intraoperative adverse events occurred in seven patients (15.2%) in the control group and in seven patients (14.3%) in the study group (difference −0.9%; 95% c.i. −16.1 to 13; *P* = 0.831).

The early postoperative complication rate was 21.7% and 34.7% in the control and study groups, respectively (difference 13.0%; 95% c.i. −5.4 to 30.5; *P* = 0.564). In the control group, all complications were classified as Clavien–Dindo grade I/II, whereas in the study group three patients (6.1%) had a complication classified as Clavien–Dindo grade IIIa/b. The study group had a mean CCI of 6.09, compared with a mean CCI of 2.06 in the control group (difference 4.03; 95% c.i. 0.23 to 7.83; *P* = 0.047). The median CCI was 0 in both the study group and control group (difference 0; 95% c.i. −1 to 0). The postoperative bleeding that required reintervention in the study group came from the stapling line.

The mean operative time was 161 minutes for patients undergoing CC and 153 minutes for patients without CC. The median blood loss was 20 and 10 ml in patients with and without CC, respectively. The length of hospital stay was 4 days in each group (*Table 3*).

**Table 3:** Intraoperative data and postoperative course until discharge

	RYGB		Difference†	P*
	Without cholecystectomy (*n* = 46)	With cholecystectomy (*n* = 49)		
Operative time (min), mean(s.d.)	152.7(31.16)	161(33.47)	8.3 (−4.86, 21.47)	0.332
Blood loss (ml), median (i.q.r.)	10 (10–100)	20 (10–30)	10 (−5, 10)	0.212
**Concomitant operation**	12 (26.1%)	15 (30.6%)		
Intestinal resection	1 (2.2%)	2 (4.1%)		
Small primary hernia repair	2 (4.3%)	0 (0%)		
Lysis of adhesions	3 (6.5%)	4 (8.2%)		
Liver biopsy	6 (13%)	7 (14.3%)		
Gastric wedge resection	0 (0%)	2 (4.1%)		
Port-a-cath ablation	0 (0%)	1 (2%)		
**Clinical assessment until discharge**				
Pain score (VAS) on day 1, mean(s.d.)	3.52(2.50)	3.47(2.18)	−0.05 (−1.02, 0.91)	0.522
Fever	1 (2.2%)	1 (2%)	−0.2 (−0.6, 8.8)	0.940
Vomiting	1 (2.2%)	0 (0%)	−2.2 (−11.4, 5.3)	0.289
Time to opioid discontinuation (days), median (i.q.r.)	2 (1–2)	1 (1–2)	1 (0, 1)	0.341
Time to reinitiation of nutrition (days), median (i.q.r.)	1(1–1))	1 (1–1)	0 (0, 0)	0.998
Time to liquid nutrition (days), median (i.q.r.)	1 (1–1)	1 (1–1)	0 (0, 0)	0.998
Time to mixed solid nutrition (days), median (i.q.r.)	2 (2–2)	2 (2–2)	0 (0, 0)	0.998
**Laboratory assessment**				
Haemoglobin (g/l) on day 1, mean(s.d.)	124.78(14.84)	124.71(13.2)	−0.07 (−4.3, 9.9)	0.442
CRP (mg/l) on day 1, mean(s.d.)	28.49(13.34)	27.67(18.06)	−0.8 (−22.1, 22.4)	0.988
WBC (g/l) on day 1, mean(s.d.)	10.12(2.51)	10.69(3.14)	0.6 (−1.3, 0.6)	0.427
LOS (days), median (i.q.r.)	4 (3–5)	4 (3–5)	0 (−1, 1)	0.333

Values are *n* (%) unless otherwise stated. †Values in parentheses are 95% confidence intervals. RYGB, Roux-en-Y gastric bypass; min, minutes; s.d., standard deviation; i.q.r., interquartile range; VAS, visual analogue scale; CRP, C-reactive protein; WBC, white blood cell count; LOS, length of stay. *χ² test.

### Late complications and follow-up

*Table 4* details late postoperative complications. The rate of late (> 30 days) postoperative complications was 21.7% in the control group and 22.4% in the study group (difference 0.7%; 95% c.i. −16.4 to 17.6; *P* = 0.811). One patient in the control group and three patients in the study group underwent reintervention (2.2% and 8.3%, respectively; difference 3.7%; 95% c.i. −8.3 to 15.7). The reasons for reintervention were an incisional hernia for the patient in the control group and an ileus, an internal hernia, and a fistula for the patients in the study group.

**Table 4:** Late postoperative complications

	RYGB		Difference†	P*
	Without cholecystectomy (*n* = 46)	With cholecystectomy (*n* = 49)		
Postoperative complication (30 days–18 months)	10 (21.7%)	11 (22.4%)	0.7 (−16.4, 17.6)	0.811
**Clavien–Dindo I**	2 (4.3%)	1 (2%)	−2.3 (−6.4, 5.1)	0.496
Dysphagia with malnutrition	2	0		
Ureteral lithiasis	0	1		
**Clavien–Dindo II**	7 (15.2%)	6 (12.2%)	−3.0 (−5.2, 6.8)	0.845
Urinary tract infection	6	0		
Renal lithiasis	1	2		
Pneumonia	0	1		
Anastomotic ulcer	0	2		
Acute renal failure	1	1		
**Clavien–Dindo IIIa**	0 (0%)	1 (2%)	2.0 (−5.6, 6.5)	0.340
Renal lithiasis	0	1		
**Clavien–Dindo IIIb**	1 (2.2%)	3 (6.1%)	3.9 (−8.2, 16.1)	0.360
Incisional hernia	1	0		
Fistula	0	1		
Internal hernia	0	1		
Ileus	0	1		
Clavien–Dindo IVa	0 (0%)	0 (0%)		
Clavien–Dindo IVb	0 (0%)	0 (0%)		
Clavien–Dindo V	0 (0%)	0 (0%)		
CCI score	3.92	5.4	1.48 (−2.48, 5.44)	0.466
Reintervention	1 (2.2%)	3 (6.1%)	3.7 (−8.3, 15.7)	0.360

Values are *n* (%) unless otherwise stated. †Values in parentheses are 95% confidence intervals. RYGB, Roux-en-Y gastric bypass; CCI, Comprehensive Complication Index. *χ² test.

**Fig. 2:**
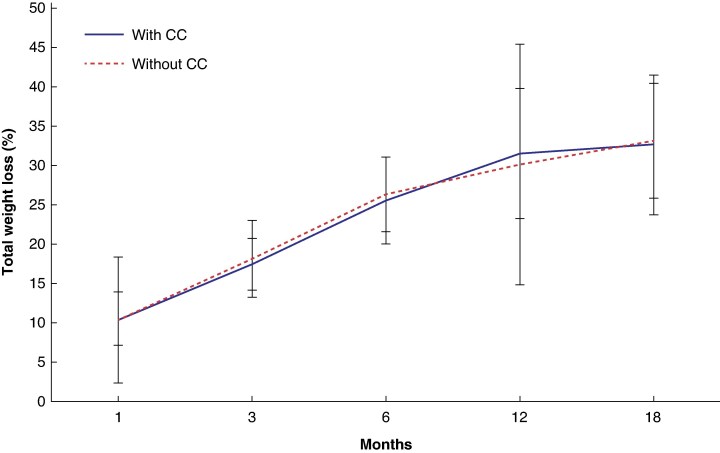
Total weight loss during follow-up Data show the mean ± standard deviation. CC, cholecystectomy.

The evolution of weight, BMI, and total weight loss is detailed in *Table S1* and *Fig. 2*. The EQ-5D™ scores, measuring quality of life, in the control and study groups were 1 and 0.98, respectively, at 1 month (difference −0.02; 95% c.i. −0.05 to 0.00; *P* = 0.897) and 0.97 and 0.97, respectively, at 12 months (difference 0; 95% c.i. −0.03 to 0.03; *P* = 0.998). The pain score at 1 month was 0 in both groups (difference 0; *P* = 0.998). The pain scores at 3, 6, 12, and 18 months are presented in *Table S1*.

### Biliary events

During the study period, three patients (6.5%) in the control group and one patient (2%) in the study group experienced one or more biliary events (*Table 5*).

**Table 5:** Biliary events during follow-up

	RYGB		Difference†	P*
	Without cholecystectomy (*n* = 46)	With cholecystectomy (*n* = 49)		
**Biliary events**	3 (6.5%)	1 (2%)	−4.5 (−14.0, 4.5)	0.248
Symptomatic gallstone	1 (2.2%)	0 (0%)	−2.2 (−11.4, 5.3)	
Gallstone migration	1 (2.2%)	1 (2%)	−0.1 (−9.6, 8.8)	
Cholecystitis	0 (0%)	0 (0%)	0 (−7.8, 7.3)	
Choledocholithiasis	0 (0%)	0 (0%)	0 (−7.8, 7.3)	
Cholangitis	0 (0%)	0 (0%)	0 (−7.8, 7.3)	
Biliary pancreatitis	0 (0%)	0 (0%)	0 (−7.8, 7.3)	
Symptomatic polyp	1 (2.2%)	0 (0%)	−2.2 (−11.4, 5.3)	
**Biliary interventions**	2 (4.4%)	0 (0%)	−2.0 (−10.2, 6.3)	0.128
Cholecystectomy	2 (4.4%)	0 (0%)	−4.3 (−14.6, 3.2)	
ERCP	0 (0%)	0 (0%)	0 (−7.8, 7.3)	
**Ultrasound at 18 months**				
Sludge	6 (15%)			
Gallstones	4 (10%)			
Polyp	1 (2.5%)			
Cholecystitis	0 (0%)			
Enlargement of main biliary duct	0 (0%)			

Values are *n* (%) unless otherwise stated. †Values in parentheses are 95% confidence intervals. RYGB, Roux-en-Y gastric bypass; ERCP, endoscopic retrograde cholangiopancreatography. *χ² test.

In the control group, one patient presented with gallstone migration at the 6-month follow-up and another patient developed symptoms related to a gallbladder polyp combined with sludge at 12 months. Both patients underwent cholecystectomy without postoperative complications. A third patient experienced biliary colic at 18 months, which was managed conservatively at the patient’s request.

In the study group, one patient experienced gallstone migration at 18 months, which was treated conservatively.

The 18-month ultrasound performed in all patients in the control group who did not undergo cholecystectomy during follow-up (*n* = 40) demonstrated the presence of sludge in six patients (15%), gallstones in four (10%), and a polyp in one (2.5%). Patients were not blinded to the results.

### Resolution of obesity-related complications

*Table S2* presents obesity-related complications at baseline and their resolution at the 18-month follow-up. In the control and study groups, respective remission rates were 78% and 75% for diabetes, 53% and 48% for hypertension, 88% and 75% for dyslipidaemia, 58% and 69% for sleep apnoea syndrome, and 88% and 89% gastro-oesophageal reflux.

## Discussion

This pilot trial investigated the impact of prophylactic CC at the time of RYGB in patients without gallstones on preoperative ultrasound. The results highlighted a slower-than-expected patient accrual, yet demonstrated the feasibility of obtaining an almost complete follow-up, providing the basis for a large randomized confirmatory trial. In addition, this study suggests that the mean 30-day CCI is higher when CC is performed. Finally, the incidence of biliary events during follow-up in patients without CC was relatively low (6.7%), with an even lower incidence of biliary interventions (4.4%). Together, the results of this preliminary analysis may add some weight to the argument against routine prophylactic CC in patients without preoperative evidence of gallstones, although the results should be interpreted with caution given the study’s small sample size and limited power.

In the scientific literature, disagreement remains about whether prophylactic cholecystectomy should be performed during RYGB^17^. In essence, surgeons try to balance the risk of complications associated with CC against the risk of future biliary complications. One possible motivation behind routine prophylactic cholecystectomy is the concern over subsequent severe biliary complications that may require endoscopic retrograde cholangiopancreatography. Indeed, endoscopic retrograde cholangiopancreatography is technically challenging in patients with modified anatomy after RYGB, because endoscopic access to the excluded stomach is impossible. However, a meta-analysis^9^ on patients receiving RYGB without CC reported a very low incidence of common bile duct stones (CBDS) and biliary pancreatitis following RYGB (0.2%). Similarly, in a large nationwide study by Marciniak *et al*.^5^, the rate of postoperative pancreatitis was 0.1%, with a 0.08% rate of CBDS. Consistent with these findings, no biliary pancreatitis or CBDS was observed in the present trial. Furthermore, emerging techniques such as endoscopic ultrasound-directed transgastric endoscopic retrograde cholangiopancreatography may improve the management of CBDS in the future^18^.

Another argument in favour of routine prophylactic CC is the risk of complications in RYGB patients undergoing interval cholecystectomy. Some studies report higher postoperative complication rates in these patients *versus* patients without previous RYGB^19,20^, likely due to more severe biliary disease (that is, acute cholecystitis or biliary pancreatitis) prompting interval cholecystectomy. However, these results contrast with other studies, such as that by Marciniak *et al*.^5^, who reported a 1.1% complication rate with interval cholecystectomy. Given the small number of patients needing biliary interventions during follow-up, the present analysis offers few new insights in this regard, although none of the patients undergoing interval cholecystectomy experienced postoperative complications.

Conversely, the potentially increased risk of postoperative complications is an argument against prophylactic CC. Although some studies^21,22^ have reported similar safety profiles regardless of whether cholecystectomy is performed, others^5,6,19,23^ highlight increased risks for patients undergoing MBS with concomitant procedures. The present pilot trial seems to align with the latter findings, in that the mean CCI was higher in patients receiving CC. In addition, the composite endpoint of intraoperative adverse events and early postoperative complications also appeared to increase with CC (44% *versus* 27%), although the difference was not statistically significant. Interestingly, this difference seems to be mainly driven by an increase in postoperative complications in patients receiving CC, because the rate of intraoperative adverse events was similar between groups. These preliminary observations require verification in a larger, adequately powered randomized trial. Further investigation using standardized definitions of complications would be invaluable in informing clinical practice.

One limitation of the present study is the prolonged patient enrolment period, which may partly be attributable to the reduced surgical activity during the COVID-19 pandemic. The significant number of patients who declined participation should also be taken into account when designing a larger randomized trial. In addition, the study’s original protocol was revised, and a feasibility outcome was later introduced as a co-primary endpoint. However, this modification was made independent of the trial results. Moreover, because patients were not blinded to the findings of the follow-up ultrasound, some of the reported symptoms may have been influenced by knowledge of the presence of gallstones. Future randomized trials should consider the need for blinding regarding the ultrasound results. A further limitation of this study is the potential for concealment issues during the randomization process due to the use of envelopes. If the envelopes were not sufficiently opaque or secure, there is a risk that researchers could have influenced the allocation process, potentially introducing bias into the study results. The relatively short follow-up of 18 months, which prevents drawing definite conclusions about subsequent biliary events over the long term, represents another significant limitation, even though most biliary events typically occur during the period of maximum weight loss in the first postoperative year^24^. Considering the high rate of follow-up achieved in this study, a future randomized trial could extend follow-up to 5 years. Finally, the feasibility analysis did not include qualitative research with patients and specialists, which should be considered in a future trial. Because this is a pilot trial aiming to provide data for further investigation, the confidence intervals were not adjusted for multiple comparisons. Given its design, this study allows for hypothesis generation, but a confirmatory trial is needed to draw definitive conclusions.

In conclusion, the debate over performing concomitant cholecystectomy during RYGB remains unresolved. These preliminary findings underscore the need for a large randomized trial and demonstrate its feasibility.

## Supplementary Material

zrag092_Supplementary_Data

## Data Availability

Data, including deidentified participant data, are available upon request from the corresponding author.
