# Supplementary material for: Robotic gastric Roux-en-Y bypass with or without concomitant cholecystectomy: pilot open label randomized trial
Source: BJS Open. 2026 Aug 18;10(4):zrag092. doi: 10.1093/bjsopen/zrag092 (PMC13482983; doi:10.1093/bjsopen/zrag092)
Supplement: Supplementary file 1 [file zrag092_supplementary_data.docx]

**Robotic gastric Roux-en-Y bypass with or without concomitant cholecystectomy: Pilot open label randomised trial**

Marie Burgard, Marco Bonino, Marion Dietrich, Pouya Iranmanesh, Michael Racine, Guillaume Giudicelli, Stefan P. Mönig, C. Toso, Minoa K. Jung

Department of Visceral Surgery, HUG- Hôpitaux Universitaires de Genève, CH-1205 Genève

**Corresponding author.**

Minoa K. Jung

Rue Gabrielle-Perret-Gentil 4, 1211 Geneva 14, Switzerland

ORCID ID: 0000-0003-2352-4774

**Supplementary Materials - Index**

| **Supplementary Figures and Tables** |  |
| --- | --- |
| Supplementary Table 1: Follow-up data | *page 2* |
| Supplementary Table 2: Resolution of obesity related complications  **Supplementary Appendixes**  CONSORT checklist  Initial trial protocol | *page 3-4*    *pages 5-8*  *pages 9-36* |

**Supplementary Figures and Tables**

Supplementary Table 1: Follow-up data

|  | **RYGB 2**  **without cholecystectomy**  **n = 46** | **RYGB with**  **cholecystectomy**  **n = 49** | **Difference**  **(95% CI)** | **P-Value** |
| --- | --- | --- | --- | --- |
| **Baseline**  Weight, kg  BMI  5Q-5D  EVA | 121.73 (22.77)  43.64 (5.42)  0.94 [0.88 to 0.98]  0 [0 to 3] | 114.98 (15.66)  42.58 (5.15)  0.92 [0.86 to 0.98]  0 [0 to 2.3] |  |  |
| **1 month FU**  Weight, kg  BMI  % TWL  EQ-5D  EVA | 109.51 (23.13)  39.3 (6.3)  10.3 (8)  1[0.96 to 1]  0 [0 to 0] | 103.2 (14.67)  38 (4.9)  10.6 (3.4)  0.98 [0.94 to 1]  0 [0 to 0] | -6.3 (-14.4 to 1.8)  -1.2 (-3.5 to 1.1)  0.3 (-2.9 to 2.3)  -0.02 (-0.05 to 0.00)  0 (0 to 0) | 0.167  0.198  0.265  0.897  0.998 |
| **3 months FU**  Weight, kg  BMI  % TWL  EVA | 99.66 (19.8)  35.7 (5.2)  18.1 (4.9)  0 [0 to 0] | 96.13 (13.85)  35.3 (5)  17.4 (3.3)  0 [0 to 0] | -3.5 (-10.8 to 3.8)  -0.4 (-2.5 to 1.8)  -0.8 (-1 to 2.6)  0 (na) | 0.420  0.567  0.618  0.998 |
| **6 months FU**  Weight, kg  BMI  % TWL  EVA | 90.14 (18.78)  32.2 (4.9)  26.3 (4.8)  0 [0 to 0] | 85.85 (13.85)  31.9 (4.9)  25.4 (5.5)  0 [0 to 0] | -4.3 (-11.2 to 2.6)  -0.3 (-2.4 to 1.7)  - 0.9 (-1.3 to 3)  0 (0 to 0) | 0.277  0.624  0.543  0.998 |
| **12 months FU**  Weight, kg  BMI  % TWL  EQ-5D  EVA | 84.48 (20.98)  30.4 (6.8)  30.2 (15.3)  0.97 (0.07)  0 [0 to 0] | 79.92 (15.85)  29.4 (5.2)  31.4 (8.3)  0.97 (0.06)  0 [0 to 0] | -4.6 (-12.4 to 3.3)  -1 (-3.6 to 1.5)  1.2 (-6.4 to 4)  0 (-0.03 to 0.03)  0 (na) | 0.264  0.342  0.441  0.998  0.998 |
| **18 months FU**  Weight, kg  BMI  % TWL  EVA | 80.42 (16.83)  29 (4.2)  33.3 (7.3)  0 [0 to 0]) | 78.31 (14.69)  28.9 (5.3)  32.4 (8.9)  0 [0 to 0] | -2.1 (-8.9 to 4.7)  -0.1 (-2.1 to 2)  0.8 (-2.7 to 4.3)  0 (na) | 0.570  0.764  0.531  0.998 |

All data are expressed in numbers n (%), the weight, body mass index (BMI) and total weight loss (TWL) are expressed in mean (standard deviation, SD), the quality of life (5Q-5D) and pain score (EVA= echelle visuelle analogue) are expressed in median [interquartile interval].

RYGB = Roux-en-Y Gastric Bypass, N = number, CI = confidence interval, FU = follow-up, kg = kilogram

Supplementary Table 2: Resolution of obesity related complications

|  | **RYGB without cholecystectomy**  **N = 45** | **RYGB with**  **cholecystectomy**  **n = 50** | **Difference**  **(95% CI)** | **P-Value** |
| --- | --- | --- | --- | --- |
| **Hypertension**  Remission  Improved  Unchanged  De novo  Unknown  **Type II diabetes**  Remission  Improved  Unchanged  De novo  Unknown  **Dyslipidaemia**  Remission  Improved  Unchanged  De novo  Unknown  **Sleep apnoea syndrome**  Remission  Improved  Unchanged  De novo  Unknown  **GERD**  Remission  Improved  Unchanged  De novo  Unknown | 9 (52.9 %)  4 (23.5%)  1 (5.9%)  0 (0%)  3 (17.6%)    7 (77.8%)  2 (22.1%)  0 (0%)  0 (0%)  3 (33.3%)  14 (87.5%)  1 (6.2%)  0 (0%)  0 (0%)  3 (18.7%)  11 (57.9%)  7 (36.8%)  2 (10.5%)  0 (0%)  3 (15.8%)  19 (86.4%)  2 (9.1%)  0 (0%)  1 (4.5%)  3 (13.6%) | 10 (47.6%)  3 (14.3%)  4 (19 %)  0 (0%)  6 (28.6%)    9 (75 %)  0 (0%)  0 (0%)  0 (0%)  6 (50%)  12 (75 %)  1 (8.3%)  0 (0%)  0 (0%)  6 (37.5%)  11 (68.8%)  2 (12.5%)  1 (6.2%)  0 (0%)  6 (37.5%)  17 (68%)  1 (4 %)  2 (8 %)  1 (4 %)  6 (24%) | -5.3 (-8.5 to 19.1)  -9.2 (-1.9 to 20)  13.1 (-0.03 to 25.9)  0 (0 to 0)  11 (-5.76 to 27.76)  -2.8 (-19.9 to 14.3)  - 22.1 (-34.2 to -9.9)  0 (0 to 0)  0 (0 to 0)  16.7 (-2.8 to 36.2)  -12.5 (-27.9 to 2.9)  2.1 (-8.2 to 12.5)  0 (0 to 0)  0 (0 to 0)  18.8 (1.2 to 36.4)  -14.3 (-30.2 to 1.6)  -4.3 (-15.5 to 6.9)  0 (0 to 0)  21.7 (4.6 to 38.8)  -18.4 (2.1 to 34.8)  -5.1 (-4.9 to 15.1)  8 (0.5 to 15.5)  -0.5 (-7.6 to 8.6)  10.4 (-25.9 to 5.1) | 0.756  0.469  0.217  0.998  0.341  0.231  0.231  0.998  0.998  0.341  0.916  0.916  0.998  0.998  0.341  0.157  0.178  0.773  0.998  0.345  0.631  0.578  0.138  0.998  0.415 |
| **Preexisting Type II diabetes**  **Fasting glucose level,** mmol/l  Baseline  18 months  **Hb1ac level,** %  Baseline  18 months | 9 (20%)  7.1 (1.7)  5 (0.6)  6.5 (0.8)  5.3 (0.4) | 12 (24%)  7.3 (2.7)  5.5 (1.5)  6.6 (1)   5.8 (1.5) | 0.2 (-1.7 to 2.2)  0.5 (-0.5 to 1.5)  0.1 (-0.7 to 0.9)  0.5 (0.4 to 1.5) | 0.786  0.189 |
| **Preexisting dyslipidaemia**  **Total cholesterol,** mmol/l  Baseline  18 months  **HDL cholesterol,** mmol/l  Baseline  18 months  **LDL cholesterol,** mmol/l  Baseline  18 months  **Triglycerides,** mmol/l  Baseline  18 months | 16 (35.6%)  5.4 (0.9)  4.3 (0.9)  1.1 (0.3)  1.4 (0.3)  3.3 (1.0)  2.5 (0.8)  2.3 (1.1)  1.0 (0.4) | 16 (32%)  5.2 (1.1)  4.0 (0.6)  1.2 (0.3)  1.5 (0.4)  3.1 (1.2)  1.9 (0.7)  2.3 (1.2)  1.1 (0.6) | -0.1 (-0.9 to 0.6)  -0-3 (-0.9 to 0.3)  0.1 (-0.1 to 0.4)  0.2 (-0.1 to 0.4)  -0.2 (-1 to 0.6)  -0.5 (-1.1 to 0)  0 (-0.9 to 0.8)  0.1 (-0.3 to 0.4) | 0.556  0.442  0.189  0.703 |
|  |  |  |  |  |

All data are expressed in numbers n (%) or mean (standard deviation, SD). RYGB = Roux-en-Y Gastric Bypass, N = number, CI = confidence interval, GERD = gastro-oesophageal reflux disease.

**Supplementary Appendixes**

CONSORT checklist

|  | Section/topic | No | CONSORT 2025 checklist item description | Reported on page no. |
| --- | --- | --- | --- | --- |
|  | **Title and abstract** | | |  |
|  | Title and structured abstract | 1a | Identification as a randomised trial | 1 |
|  |  | 1b | Structured summary of the trial design, methods, results, and conclusions | 2 |
|  | **Open science** | | |  |
|  | Trial registration | 2 | Name of trial registry, identifying number (with URL) and date of registration | 4 |
|  | Protocol and statistical analysis plan | 3 | Where the trial protocol and statistical analysis plan can be accessed | 4 |
|  | Data sharing | 4 | Where and how the individual de-identified participant data (including data dictionary), statistical code and any other materials can be accessed | 4 |
|  | Funding and conflicts of interest | 5a | Sources of funding and other support (eg, supply of drugs), and role of funders in the design, conduct, analysis and reporting of the trial | 1 |
|  |  | 5b | Financial and other conflicts of interest of the manuscript authors | 1 |
|  | **Introduction** | | |  |
|  | Background and rationale | 6 | Scientific background and rationale | 3 |
|  | Objectives | 7 | Specific objectives related to benefits and harms | 3 |
|  | **Methods** | | |  |
|  | Patient and public involvement | 8 | Details of patient or public involvement in the design, conduct and reporting of the trial | 3,4 |
|  | Trial design | 9 | Description of trial design including type of trial (eg, parallel group, crossover), allocation ratio, and framework (eg, superiority, equivalence, non-inferiority, exploratory) | 3,4 |
|  | Changes to trial protocol | 10 | Important changes to the trial after it commenced including any outcomes or analyses that were not prespecified, with reason | 4 |
|  | Trial setting | 11 | Settings (eg, community, hospital) and locations (eg, countries, sites) where the trial was conducted | 3 |
|  | Eligibility criteria | 12a | Eligibility criteria for participants | 3 |
|  |  | 12b | If applicable, eligibility criteria for sites and for individuals delivering the interventions (eg, surgeons, physiotherapists) | 3 |
|  | Intervention and comparator | 13 | Intervention and comparator with sufficient details to allow replication. If relevant, where additional materials describing the intervention and comparator (eg, intervention manual) can be accessed | 4 |
|  | Outcomes | 14 | Prespecified primary and secondary outcomes, including the specific measurement variable (eg, systolic blood pressure), analysis metric (eg, change from baseline, final value, time to event), method of aggregation (eg, median, proportion), and time point for each outcome | 5 |
|  | Harms | 15 | How harms were defined and assessed (eg, systematically, non-systematically) |  |
|  | Sample size | 16a | How sample size was determined, including all assumptions supporting the sample size calculation | 5 |
|  |  | 16b | Explanation of any interim analyses and stopping guidelines |  |
|  | Randomisation: |  |  |  |
|  | Sequence generation | 17a | Who generated the random allocation sequence and the method used | 4 |
|  |  | 17b | Type of randomisation and details of any restriction (eg, stratification, blocking and block size) | 4 |
|  |  |  |  | **Reported on page no.** |
|  | Allocation concealment mechanism | 18 | Mechanism used to implement the random allocation sequence (eg, central computer/telephone; sequentially numbered, opaque, sealed containers), describing any steps to conceal the sequence until interventions were assigned | 4 |
|  | Implementation | 19 | Whether the personnel who enrolled and those who assigned participants to the interventions had access to the random allocation sequence | 4 |
|  | Blinding | 20a | Who was blinded after assignment to interventions (eg, participants, care providers, outcome assessors, data analysts) | 4 |
|  |  | 20b | If blinded, how blinding was achieved and description of the similarity of interventions | n/a |
|  | Statistical methods | 21a | Statistical methods used to compare groups for primary and secondary outcomes, including harms | 5 |
|  |  | 21b | Definition of who is included in each analysis (eg, all randomised participants), and in which group |  |
|  |  | 21c | How missing data were handled in the analysis | 5 |
|  |  | 21d | Methods for any additional analyses (eg, subgroup and sensitivity analyses), distinguishing prespecified from post hoc | 5 |
|  | **Results** | | |  |
|  | Participant flow, including flow diagram | 22a | For each group, the numbers of participants who were randomly assigned, received intended intervention, and were analysed for the primary outcome | 6 |
|  |  | 22b | For each group, losses and exclusions after randomisation, together with reasons | 6 |
|  | Recruitment | 23a | Dates defining the periods of recruitment and follow-up for outcomes of benefits and harms | 6 |
|  |  | 23b | If relevant, why the trial ended or was stopped | n/a |
|  | Intervention and comparator delivery | 24a | Intervention and comparator as they were actually administered (eg, where appropriate, who delivered the intervention/comparator, how participants adhered, whether they were delivered as intended (fidelity)) | 6 |
|  |  | 24b | Concomitant care received during the trial for each group |  |
|  | Baseline data | 25 | A table showing baseline demographic and clinical characteristics for each group | Table 1-7 |
|  | Numbers analysed,  outcomes and estimation | 26 | For each primary and secondary outcome, by group:  ● the number of participants included in the analysis  ● the number of participants with available data at the outcome time point  ● result for each group, and the estimated effect size and its precision (such as 95% confidence interval)  ● for binary outcomes, presentation of both absolute and relative effect size |  |
|  | Harms | 27 | All harms or unintended events in each group | n/a |
|  | Ancillary analyses | 28 | Any other analyses performed, including subgroup and sensitivity analyses, distinguishing pre-specified from post hoc |  |
|  | **Discussion** | | |  |
|  | Interpretation | 29 | Interpretation consistent with results, balancing benefits and harms, and considering other relevant evidence | 7,8,9 |
|  | Limitations | 30 | Trial limitations, addressing sources of potential bias, imprecision, generalisability, and, if relevant, multiplicity of analyses | 8,9 |

Citation: Hopewell S, Chan AW, Collins GS, Hróbjartsson A, Moher D, Schulz KF, et al. CONSORT 2025 Statement: updated guideline for reporting randomised trials. BMJ. 2025; 388:e081123. <https://dx.doi.org/10.1136/bmj-2024-081123>
© 2025 Hopewell et al. This is an Open Access article distributed under the terms of the Creative Commons Attribution License (<https://creativecommons.org/licenses/by/4.0/>), which permits unrestricted use, distribution, and reproduction in any medium, provided the original work is properly cited.

*We strongly recommend reading this statement in conjunction with the CONSORT 2025 Explanation and Elaboration and/or the CONSORT 2025 Expanded Checklist for important clarifications on all the items. We also recommend reading relevant CONSORT extensions. See [www.consort-spirit.org](http://www.consort-spirit.org).

Initial trial protocol

1. Introduction and Background

While morbidly obese patients might be at high risk to develop gallstones, concomitant cholecystectomy at time of gastric bypass is still debated. Clear guidelines concerning the need of concomitant cholecystectomy during gastric bypass procedure are still lacking. The

“Pratique Clinique de la Société Nationale Francaise de Gastroentérologie” sums up that they can not deliver clear recommendations concerning cholecystectomy during gastric bypass. The guidelines of the HAS (Haute Autorité de Santé de France) of 01/2015	conclude

that: “in the current stage of knowledge, no recommendations can be made regarding

prophylactic cholecystectomy during bariatric surgery.” The "Swiss Study Group for Morbid Obesity" (SMOB) which was mandated by the « Office Fe´de´ral d e la Sante´ Publique » (OFSP) le 22.1.2008 to revise the guidelines for the surgical treatment of obesity does not mention the necessity of a concomitant cholecystectomy neither.

The prevalence of gallstones in morbidly obese patients varies between 19% and 35% (1, 2). The risk of developing gallstones after rapid weight loss after bariatric surgery might even be more important. It has been shown that rapid weight loss can further enhance the risk of cholesterol cholelithiasis (

4). However, there are contradictory reports regarding	true

**Code de champ modifié**

incidence of symptomatic cholelithiasis necessitating cholecystectomy after gastric bypass. Concomitant cholecystectomy at time of gastric bypass has become rare over the last years decreasing from 26.3% to only 3.7% (5) and rate of secondary cholecystectomy after RYGB is reported in varied rates from 3% to 28% of cases (6-8). A metaanalysis of Warschkow et al including 6’048 patients reported a very low rate of 6.8% of subsequent cholecystectomy after RYGB with a main cause for the subsequent cholecystectomy of uncomplicated biliary disease (9). Worni et al evaluated data from 70’287 patients undergoing laparoscopic gastric bypass and only 9.1% underwent concomitant cholecystectomy (5). Proportion of patients who underwent concomitant cholecystectomy during the study period decreased from 26.3

**Code de champ modifié**

**Code de champ modifié**

**Code de champ modifié**

**Code de champ modifié**

% to 3.7% over the 8 year time period. Strongest predictor for concomitant cholecystectomy was the presence of gallbladder disease. Villegas et al (10) reported the additional operative time for cholecystectomy to be 20 minutes. Tucker et al (11) reported the addition of a mean operative time of 18 minutes for concomitant cholecystectomy. Intraoperative complications occurred in 2.37% of patients without concomitant cholecystectomy and in 2.86% of the procedures with concomitant cholecystectomy (5). Patients who underwent

**Code de champ modifié**

**Code de champ modifié**

**Code de champ modifié Code de champ modifié**

concomitant cholecystectomy underwent a higher number of postoperative complications (6.22% versus 5.06%, p<0.001) in terms of postoperative infections, pulmonary complications and gastrointestinal complications (5). Dorman at al confirmed a total adverse event rate of 6.6% among RYGB with concomitant cholecystectomy compared to 4.9% among RYBG alone patients (p<0.001) (12).

Recent date concerning patients with RYGB without concomitant cholecystectomy revealed that 91- 97% remain asymptomatic and never need reintervention (13, 14). Higher rates of intra- and postoperative adverse events in cases of concomitant cholecystectomy and longer operation times have convinced surgeons over time period to retain from concomitant cholecystectomy.

The gallbladder management during gastric bypass remains controversial. Three different approaches have been proposed. First, prophylactic concomitant cholecystectomy in all patients undergoing gastric bypass without preoperative ultrasound, the so far most chosen approach in our institution. Second, a selective approach, based on which cholecystectomy is only performed in case of the presence of gallstones confirmed in preoperative ultrasound. Third, no cholecystectomy is performed with optional prophylactic medication against development of gallstones (ursodeoxycholic acid). Retrospective studies were in favor of a selective approach with concomitant cholecystectomy during gastric bypass only in case of preoperatively detected gallbladder pathology by ultrasound and not to treat patients without gallbladder pathology at all versus prophylactic treatment with ursodeoxycholic acid to evit further stone development with weight loss (15,16). Ursodiol is prescribed by one third of bariatric surgeons (1). Sugerman et al (17) described a 2% rate of gallstone formation in patients receiving ursodiol after gastric bypass versus 32% in the placebo group. The development of symptoms and the need for cholecystectomy, however, were not reported in the study. Turnheer et al showed in a metaanalysis including 6’048 patients that postoperative ursodoxycholic acid therapy had no significant influence on secondary cholecystectomy rate after gastric bypass (9). An analysis of cost-effectiveness showed that the use of ursodiol was associated with an increase in cost not justifying its use in the setting of a follow up in the outpatient clinic where gallbladder related symptoms and/or stones will be detected and patients referred to secondary cholecystectomy if needed (18). Retrospective Data has shown that RYGB with concomitant cholecystectomy have a longer operation time with an increase of the rate of postoperative morbidity (5, 18).

**Code de champ modifié**

**Code de champ modifié**

**Code de champ modifié Code de champ modifié**

Warschkow et al (9) showed in their metaanalysis including 6’048 patients that if the indication for concomitant cholecystectomy during RYGB was symptomatic biliary disease, the rate of subsequent cholecystectomies was 8% while if the indication was cholecystolithiasis, the rate was reduced to 4.8%. Morbidity of a subsequent cholecystectomy in the group of RYGB without concomitant cholecystectomy at first was 1.8% (including 7’796 patients with 336 subsequent cholecystectomies). This corresponds to a risk of 0.1% for all RYGB patients with gallbladder in situ to suffer a complication during a subsequent cholecystectomy. No prospective randomized trial exists investigating the role of not performing concomitant cholecystectomy in gastric bypass in case of absence of gallbladder pathology. Retrospective data comparing the two procedures might lack in reliability and underestimate real occurrence of complications especially complications of lower degree Dindo I and II which are known to be underestimated in retrospective data collection. We would like to perform this pilot randomized trial in order to obtain data of higher reliability which will allow us to plan a future larger multicentric trial. In this pilot study, we will explore the following hypotheses:

**Code de champ modifié**

Obese Patients without presence of gallstones in preoperative Ultrasound should undergo RYGB without cholecystectomy. This strategy will decrease the incidence of adverse	events

length of the operation, the length of hospital stay as well as overall costs. This pilot study

patient and	compare it with	these	events	in	the gastric	bypass	procedure	without

metaanalysis of Warschkow et al (9) not only patients with symptomatic biliary disease but

complications for patients with known gallbladder disease. Our findings should help to find

cholecystectomy in case of untrasonographically confirmed gallbladder diseases (stones) and

decrease the operative risk and the risk of postoperative complications

.

2. Main Objective and Endpoints

The main objective of this pilot study is to show feasibility to collect peri-operative and postoperative clinical data of the study group of gastric bypass without cholecystectomy compared with the control group of gastric bypass with cholecystectomy. Our hypothesis is that the approach without cholecystectomy would be superior in terms of a decrease of perioperative adverse events and postoperative complications, as well as lenght of operation, lenght of hospital stay, overall costs with a very low risk of biliary complication in the follow up. These findings could be helpful to build a baseline for a future randomized controlled multicenter study allowing significance of these results and help orientate surgeons towards best surgical care of the gallbladder with confirmed absence of stones in the obese patient undergoing gastric bypass.

Primary endpoint:

1. composite endpoint consisting of:

a.: intraoperative adverse events (common bile duct lesion, lesion of liver, lesion of digestive tract, bleeding, conversion).

b.: postoperative complications according to Dindo/Clavien Classification

Secondary endpoints:

1. Intraoperative adverse events in detail:
   - - Common bile duct lesion
   - - Lesion of Liver
   - - Lesion of digestive tract
   - - bleeding
   - - conversion
2. Morbidity and mortality assessed by Dindo/ Clavien Classification system 3.length of operation time

4. length of hospital stay (discharge criteria defined as follows : absence of fever (T≤37.8), Neutrophils ≤12G/L, Absence of anemia, no nausea, no vomiting)
5. asymptomatic gallstones 6.symptomatic gallstones
7. cholecystitis
8. choledocholithiasis (biliary complication)
15. cholangitis (biliary complication)
16. biliary pancreatitis (biliary complication)
17. Average costs
18. Quality of life
19. Readmission
20. Re-intervention (Cholecystectomy, transgastric ERCP, biliary Revision)
    3. Patients and Methods

A total of 90 consecutive obese patients attending the Unit of Visceral Surgery

of the University Hospital of Geneva who meet the criteria for bariatric surgery and where the preoperative ultrasound confirms absence of gallstones will be recruited for the study.

A prospective open randomized pilot study design will be used. At 6 follow-up visits (at discharge, 1 ,3 ,6 ,12 and 18 months post-surgery), patients will be evaluated.

This study will be conducted in compliance with the protocol, the current version of the Declaration of Helsinki, the ICH-GCP or ISO EN 14155 as wella s all national legal and regulatory requirements, the LPth (Loi sur les produits thérapeutiques 812.21) and the OClin (Ordonnance sur les essais cliniques des produits thérapeutiques 812.214.2).

3.1. Study Duration

The expected duration of the study is 3 years. Patients will be recruited during 12 months (from July 2016 to June 2017), approximately 120 robotic gastric RYGB are performed per year at the HUG). Study period will include screening, surgical intervention and 5 follow-up visitis (1,3,6,12 and 18 months postoperatively).

3.2. Description of surgical technique

One optical trocar, three 12 mm trocars and one 5 mm trocar are placed. No additional trocar has to be placed for concomitant cholecystectomy. The intervention will start with the cholecystectomy laparoscopically. A small gastric pouch (around 20-30 cc) is created using blue cartridge staplers. A standard robotic RYGB with a 75 cm biliary limb and a 150 cm antecolic alimentary limb is performed. A hand-sewn gastrojejunal and jejunojejunal anastomois are performed, using a single layer running suture of Vicryl 2.0.

3.3. Collected Data

Data collected will include the following:

Pre-Operative demographic data and other variables will be collected at baseline and at some of the 6 follow-up points (at discharge, 1,3,6,12,18 months):

Ultrasound, Patient Demographics and pre-Operative History will be collected, including: Gender, age on admission for operation, BMI, Height, Weight, ASA score, history of abdominal surgery, co- morbidities (Type 2 diabetes requiring

Insulin or oral medication), Hypertension requiring medication, Coronary heart disease, including history of myocardial infarction, angina pectoris, coronary artery surgery Pulmonary comorbidities including history of pulmonary embolism, Sleep Apnea

Tobacco use, Medication used by patient, Systolic and diastolic blood pressure, heart rate, Fasting plasma glucose, HbA1c, laboratory findings, QOL using the EQ-5D-5L questionnaire (21)

Preoperative routine work-up includes a physical examination, vital parameters, laboratory analyses (hematology, chemistry and HbA1c), sleep apnea evaluation with Polygraphia, abdominal ultrasound, endoscopy of the stomach, pulmonary function, preoperative anesthesia consultation and psychological evaluation.

The results of these routine tests will be used to assess the patients’eligibility to participate to the proposed pilot study.

Patients information documents will be provided during the screening period (1-6 months before operation), to ensure patients have enough time to read and understand the documents.

Intra-Operative Assessment: Intra-Operative data such as complications (organ lesion, bleeding, conversion), operative time, docking times, estimated blood loss will be collected

Post-Operative Assessment: From surgery to discharge, the following patient information will be collected: complications according to Clavien/Dindo Classification system, re- operation, re-admission, length of hospital stay

Cost Analysis: Cost Data will be collected from the Administrations Database which collects cost drivers according to REKOLE. The REKOLE method for hospital cost analysis is the national standard for hospital cost analyses. All cost points including time of medical staff, material, drugs, hotel sector, operating room and others are captured automatically.

Follow-Up Assessments: Patients will come to routine controls in the outpatient clinic at 1,3,6,12 and 18 months after surgery to collect the following information:

1month Follow-up

- Postoperative complications according Dindo/Clavien Classification
- re-operations, re-admissions
- Clinical and Laboratory findings: creatinine, blood count, liver function tests (AST, ALT, gGT, alkaline phosphatase, bilirubine)
- QOL using the EQ-5D-5L questionnaire

3 month Follow-up

- Postoperative complications according Dindo/Clavien Classification
- Re-operations, re-admissions
- Clinical and Laboratory findings: creatinine, blood count, liver function tests (AST, ALT, gGT, alkaline phosphatase, bilirubine)

6 month Follow-up

- Postoperative complications according Dindo/Clavien Classification
- re-operations, re-admissions
- Clinical and Laboratory findings: creatinine, blood count, liver function tests (AST, ALT, gGT, alkaline phosphatase, bilirubine)
- QOL using the EQ-5D-5L questionnaire

12 month Follow-up

- Postoperative complications according Dindo/Clavien Classification
- re-operations, re-admissions
- Clinical and Laboratory findings: creatinine, blood count, liver function tests (AST, ALT, gGT, alkaline phosphatase, bilirubine)

18 month Follow-up

- Postoperative complications according Dindo/Clavien Classification
- Ultrasound
- re-operations, re-admissions
- Clinical and Laboratory findings: creatinine, blood count, liver function tests (AST, ALT, gGT, alkaline phosphatase, bilirubine)
- QOL using the EQ-5D-5L questionnaire

Please refer to the data collection forms/Case report forms for complete details of perioperative data to be collected.

4. Study
4.1. **Study Design**

We suggest performing a prospective randomized pilot trial comparing RYGB without concomitant cholecystectomy with RYGB with concomitant cholecystectomy for patients with absence of gallstones in preoperative ultrasound. Patients with ultrasonographically confirmed gallstones in the gallbladder undergo concomitant cholecystectomy at time of RYGB and are excluded from the study.

**Preoperative**

**Ultrasonography before RYGB**

positive

ultrasonography

**negative**

**ultrasonography randomized in two groups**

concomitant CCK

**A: no CCK**

**B: concomitant**

**CCK**

follow up by clinical

exam and lab at 1, 6,

12,18 mts

questionnaire at 6 months; Ultrasound and questionnaire at 18 months

follow up by clinical

exam, and lab at 1, 6,

12,18 mts.

questionnaire at 6 and 18 months

4.2. Randomization

Randomization and management strategies:

Patients who meet the inclusion criteria will be randomized according to a randomization list generated by the CRC of the University Hospital of Geneva. The randomization envelops will be opened by the study staff at the appropriate time prior to the scheduling of gastric bypass after subject selection. The randomization assignment insert will contain information specifying whether the cholecystectomy will be performed or not during gastric bypass procedure.

4.3. Study Size

Since this study is a pilot study, no sample size calculation was done. The recruitment capacity over a 1-year period is 90 participants. With a sample size of 45 participants in each arm, the prevalence of the composite outcome (adverse events and complications) will be assessed with a precision (half length of the 95% confidence interval) of +/-0.09 for a prevalence of 0.15 and +/-0.13 for a prevalence of 0.25. This sample size allows to explore the grade of complications and to provide information for a sample size calculation for a confirmatory trial.

4.4. Data Analysis

All endpoints will be described by arm. Prevalence of the composite endpoint (primary endpoint) and binary secondary endpoints will be reported with exact 95% confidence interval. Continuous endpoints will be summarized by the mean (with 95% confidence interval) or by the median (with interquartile interval).

4.5. Study Duration

The patient enrollment, data collection and data entry including the follow-up period for this study is anticipated to be completed in approximately 30 months. Data analysis is anticipated to be completed in approximately 3 month. The duration of the entire study is expected to be approximately 36 months.

5. Subject Eligibility

During the screening period, the clinical and laboratory exams conducted in routine clinical care will take place. Eligible patients will be recruited from the Principle Investigator’s or one of the Co-Investigator’s clinical practice. The screening requirements will include evaluation

of clinical findings in the outpatient setting during the pre-operative period utilizing routine clinical evaluation. All patients who are eligible based on the inclusion and exclusion criteria of this study will be offered enrollment into this study.

Subjects considered eligible for study inclusion:

5.1. Inclusion criteria:
     - Patients must meet the criteria of the Swiss society for the study of morbid obesity (SMOB) to undergo gastric bypass (19):
       - BMI ≥ 35 kg/m^2^
       - Having followed a 2 year adequate program of weight loss without success or a 1 year program in case of “superobesity” (BMI ≥ 50 kg/m^2^)
       - Consent to multidisciplinary follow up for 5 years
     - Preoperative ultrasound without presence of gallstones or polyps
     - Age ≥ 18 years
5.2. Exclusion criteria :
     - Patients which have a contra-indication for gastric bypass according to the criteria of the Swiss society for the study of morbid obesity (SMOB) to undergo gastric bypass (19):
       - Pregnancy
       - Kidney failure (creatinine ≥ 300mmol/l, GFR < 30ml/min) without dialysis
       - Cirrhosis Child B/C
       - Ulcerative Colitis
       - Pulmonary embolism or deep venous thrombosis during the last 6 months
       - Psychiatric contra indications
       - Drug abuse (alcohol, cannabis, opioids) during the last 6 months
     - Presence of gallstones on preoperative Ultrasound (which will require concomitant cholecystectomy)
     - Patients with clinical diagnosis of cholecystitis defined as right upper quadrant abdominal pain, radiological signs of cholecystits and laboratory signs of infection or

gallstone migration, defined as right upper quadrant and abnormal liver function tests

(any increase in AST, ALT, alkaline Phosphatase, GGT and/or bilirubin).

- Previous bariatric surgery other than gastric banding
- Open bypass procedure
- Medical conditions preventing informed consent

Preoperative routine work-up includes a physical examination, vital parameters, laboratory analyses (hematology, chemistry and HbA1c), sleep apnea evaluation with Polygraphia, abdominal ultrasound, endoscopy of the stomach, pulmonary function, preoperative anesthesia consultation and psychological evaluation.

The results of these routine tests will be used to assess the patients’eligiibility to participate to the proposed pilot study.

5.3. Informed Consent

Study specific Informed Consent will be obtained in writing from each subject before any procedure specific to the clinical investigation is performed. Prior to this, the investigator or his/her delegate must inform each patient of the objectives, benefits, risks and requirements imposed by the study. The patient will be provided with an information and consent form in clear, simple language. He/she must be allowed ample time to inquire about details of the study and to decide whether or not to participate to the study.

Two original information and consent forms must be completed, dated and signed personally by the patient and by the person responsible for collecting the informed consent. The patient will be given one signed original information and consent form. The second original will be kept by the investigator.

5.4. Subject Discontinuation

A subject’s participation in any clinical study is voluntary and the subject has a right to withdraw at any time without penalty or loss of benefit.

5.5. Ethics Committee

The study protocol, the participant information and consent form, and the list of investigators will be submitted to the Ethical Committee (CCER, Genève) by the Principal

Investigator. The study will not start before written approval by the CCER has been obtaines, the local regulatory requirements have been complied with.

6. Risks and benefits

The study is not anticipated to impose additional risks to the patients other than risks commonly associated with the applied standard surgical procedures and approaches. The surgeons will practice standard of care as usual. The procedure without cholecystectomy concerning the study group does not include a higher operative risk than the former standard of care of our institution where the cholecystectomy was a standard concomitant procedure of the gastric bypass.

Specific risks of the study group and the control group are as follows:

- Study group: Without concomitant cholecystectomy: development of gallstones with or without associated cholecystitis detected in postoperative clinical, biological and ultrasonographic follow up and treated by subsequent cholecystectomy. A subsequent cholecystectomy will be performed in case of symptomatic gallstones or cholecystitis. This procedure (cholecystectomy) will be performed minimal invasively and is generally not considered as a complication and can be performed in safe circumstances with less technical difficulties in patients after weight loss. The risk of biliary complications such as development of choledocholithias, cholangitis or biliary pancreatitis is described as very low 0.2% (Warschkow et al) and will be treated by transgastric or retrograde ERCP. Such a biliary complication is very rare and can occur in both procedures, with and without concomitant cholecystectomy.
- Control group: With concomitant Cholecystectomy: intra- and postoperative bleeding of the liver bed or the cystic artery, postoperative biliary leaks, lesion of hepatic artery, loss of gallstones, abcesses, common bile duct lesions, duodenal and gastric lesions, increase of operation time.
- ERCP which is indicated in the case of choledocholithaisis, cholangitis or biliary pancreatitis can have the following complications: papillary bleeding, cholangitis, pancreatitis, intestinal perforation

The potential benefit of the procedure of gastric bypass without concomitant cholecystectomy could be a shorter length of operation, a slight decrease of perioperative

adverse event and postoperative complication without relevant increase of the risk of a future biliary complication.

Any AE (Adverse Event), SAE (severe Adverse Event) and SUSAR (Suspected Unexpected Serious Adverse Reaction) will be monitored in the attached file (page at the end of the case report form) and transmitted according to the usual procedure:

- To the “promoteur” within 24 hours
- Within 15 days to the Ethical Committee in cases of SUSARs (within 7 days if death)
- In the Ethical Committee safety annual report for Simple SAE ( within 7 days if death)

7: Data Handling and record keeping

**7.1 Study Data**

The investigator will be responsible for reporting the patient’s personal details and ID number in an electronic subject identification list. To ensure confidentiality, this electronic list will be kept in a separate file and only the investigator and his/her co-investigators will have access to this file. The signed informed consent forms will be locked cupboard. The investigator must agree to provide access on site to the auditor, representatives from the Ethics Committee and/or the Regulatory Authorities.

A Case Report Form (CRF) will be designed to record all the data described by the protocol and collected by the investigator. A CRF will be completed for each participant.

The investigator, or the designated person from his/her team, agrees to complete the CRF sheets, at each participant visit, and all other documents provided by the sponsor (e.g. documents relating to the treatment management and laboratory samples). The investigator will ensure accuracy, completeness, legibility and timeliness of the data reported in the CRF and in all required documents.

7.2. Data Collection

The Data Collection Forms are the primary data collection instruments for this study. Primary data collection will be based on source documents from hospital and/or office charts, laboratory and exam charts and will be performed clearly and accurately by the lead study coordinator/designee on the Case Report Forms and study questionnaires. Results from laboratory investigations and from ultrasound will not be transribed on the CRF, but kept

separately. The data from the Data Collection forms may be entered into an electronic clinical database system. The Study investigator /study coordinator will be responsible to make sure that the patient IDs related to the study matches on the case report forms.

Data collection and data entry will be the responsibility of the Primary Investigator.

7.3. Data Management

All enrolled patients will be assigned with a unique study identifier stripped from all personal information and all potential identifiers. The study identifier will be used to label all case report forms and data during the collection, storage and analysis. Information about the study subjects will be kept confidential and Patient confidentiality will be strictly maintained. Access to patient records and history will be limited to the study investigators and the study nurse. All data will be entered and stored in a database and data validation will be carried out by the study monitor.

7.4. Archiving

The investigator will keep all information relevant to the study for at least 10 years after the end of the study. As required by Swiss regulations, the investigator must arrange for the retention of the subject identification codes for a sufficient period of time to permit any medical follow-up which may be warranted. It must be possible to identify each trial subject by name against subject and product container identification codes, treatment assignment, and the CRF. Subject files and other supporting data must be kept for at least 10 years after the end of the study.

The sponsor must make appropriate arrangements for the retention of all other essential documentation pertaining to the clinical trial in a form which can be retrieved for future reference. The protocol, documentation, approvals and all other essential documents related to the trial, including certificates that satisfactory audit and inspection procedures have been carried out, must be retained by the sponsor. Data on adverse events must always be included. All data and documents should be made available if requested by relevant authorities

7.5. Handling of Blood Samples

The blood samples will be analysed immediately as it is routine for gastric bypass patients and not stored afterwards.

7.6. Insurance

Clinical trials insurance will be provided by the University Hospitals of Geneva to cover all pecuniary consequences of public liability that may incur under the terms of common law on account of damage which may result from the clinical trial.

8. Investigator Selection

Participating Investigators are qualified surgeons experienced with surgical management of obese patients undergoing bariatric surgery with concomitant cholecystectomy.

8.1. List of Participating Investigators

Principal Investigator:

- Jung Minoa, Médecin Adjointe, Département de Chirurgie, Hôpitaux Universitaires de Genève
